# Urokinase-Type Plasminogen Activator Receptor (uPAR) Expression and [^64^Cu]Cu-DOTA-AE105 uPAR-PET/CT in Patient-Derived Xenograft Models of Oral Squamous Cell Carcinoma

**DOI:** 10.1007/s11307-023-01858-x

**Published:** 2023-09-25

**Authors:** Mads Lawaetz, Tina Binderup, Anders Christensen, Karina Juhl, Giedrius Lelkaitis, Eva Lykke, Line Knudsen, Christian von Buchwald, Andreas Kjaer

**Affiliations:** 1 Department of Otolaryngology, Head and Neck Surgery and Audiology, Rigshospitalet, Copenhagen University Hospital, Copenhagen, Denmark; 2 Department of Clinical Physiology, Nuclear Medicine and PET and Cluster for Molecular Imaging, Copenhagen University Hospital - Rigshospitalet & Department of Biomedical Sciences, University of Copenhagen, Copenhagen, Denmark; 3 Department of Pathology, Rigshospitalet, Copenhagen University Hospital, Copenhagen, Denmark

**Keywords:** Urokinase-type plasminogen activator receptor, Patient-derived xenograft models, PET/CT, ^64^Cu-DOTA-AE105, Oral squamous cell carcinoma

## Abstract

**Purpose:**

[^64^Cu]Cu-DOTA-AE105 urokinase-type plasminogen activator receptor (uPAR)-PET/CT is a novel and promising imaging modality for cancer visualization, although it has not been tested in head and neck cancer patients nor in preclinical models that closely resemble these heterogenous tumors, i.e., patient-derived xenograft (PDX) models. The aim of the present study was to establish and validate oral squamous cell carcinoma (OSCC) PDX models and to evaluate [^64^Cu]Cu-uPAR-PET/CT for tumor imaging in these models.

**Procedures:**

PDX flank tumor models were established by engrafting tumor tissue from three patients with locally advanced OSCC into immunodeficient mice. [^64^Cu]Cu-DOTA-AE105 was injected in passage 2 (P2) mice, and [^64^Cu]Cu-uPAR-PET/CT was performed 1 h and 24 h after injection. After the last PET scan, all animals were euthanized, and tumors dissected for autoradiography and immunohistochemical (IHC) staining.

**Results:**

Three PDX models were established, and all of them showed histological stability and unchanged heterogenicity, uPAR expression, and Ki67 expression through passages. A significant correlation between uPAR expression and tumor growth was found. All tumors of all models (*n*=29) showed tumor uptake of [^64^Cu]Cu-DOTA-AE105. There was a clear visual concordance between the distribution of uPAR expression (IHC) and [^64^Cu]Cu-DOTA-AE105 uptake pattern in tumor tissue (autoradiography). No significant correlation was found between IHC (H-score) and PET-signal (SUV_max_) (*r*=0.34; *p*=0.07).

**Conclusions:**

OSCC PDX models in early passages histologically mimic donor tumors and could serve as a valuable platform for the development of uPAR-targeted imaging and therapeutic modalities. Furthermore, [64Cu]Cu-uPAR-PET/CT showed target- and tumor-specific uptake in OSCC PDX models demonstrating the diagnostic potential of this modality for OSCC patients.

**Supplementary Information:**

The online version contains supplementary material available at 10.1007/s11307-023-01858-x.

## Introduction

Head and neck cancer is the seventh most prevalent malignancy worldwide, with more than 850,000 new cases per year [1]. Of these, cancer of the oral squamous cell carcinoma (OSCC) represents the most frequent type. Despite advancement in treatment, the prognosis in the recent decades has stayed poor with a 5-year overall survival of approximately 45–65 % [2, 3]. One of the major challenges in OSCC is to identify metastases to regional lymph nodes especially in patients with early-stage disease, which is reflected in a high number of patients with occult metastases (20–30%) [4–6]. Current non-invasive imaging techniques like computerized tomography (CT), magnetic resonance imaging (MRI), or ^18^F-flouro-deoxy-glucose positron emission tomography (^18^F-FDG-PET) lack the ability to accurately identify small nodal tumor deposits, and therefore, patients with early-stage disease without clinical metastases are recommended removal of regional lymph nodes either as sentinel node or elective neck dissection [7, 8]. Accurate staging and effective treatment are essential for improving the prognosis for patients with OSCC. Consequentially, there is a clinical need to improve existing OSCC diagnostic approaches. In this search, targeted molecular imaging is expected to play an important role. Tumor-targeted molecular imaging enables tumor-specific visualization and has the potential to identify patients who may benefit from targeted treatment (e.g., radionuclide therapy) as well as monitoring treatment effect [9]. Numerous biomarkers have been examined as targets for PET imaging of head and neck cancer. These biomarkers encompass integrin αvβ6 [10], integrin αvβ3 [11], epidermal growth factor receptor (EGFR) [12], and poly(ADP-ribose)polymerase-1 (PARP-1) [13]. However, none of these has been applied in clinical practice.

The urokinase-type plasminogen activator receptor (uPAR) is a cell membrane receptor converting plasminogen to plasmin, thereby activating several proteases leading to degradation of extracellular matrix, which facilitates cancer cell invasion. uPAR has been shown to be involved in many aspects of tumor development including tumor invasion and metastasis [14, 15]. The utilization of uPAR as a biomarker for PET imaging of OSCC is a topic of significant interest. One of the notable advantages associated with uPAR is its significant expression in OSCC, observed in primary tumors, lymph node metastases, and recurring tumor tissue. Moreover, uPAR is highly expressed along the invasive front within tumors and in tumor-related activated stromal cells, while its expression in normal tissue is limited [14, 16–18]. Thus, uPAR is an attractive imaging and therapeutic target. uPAR has in clinical phase II studies been investigated as a nuclear medicine-based molecular imaging target for PET in different cancers including prostate [19], neuroendocrine [20], and head and neck [21], where it has shown a significant prognostic value. All clinical uPAR-PET studies have used the peptide (AE105), with high affinity to uPAR, radiolabelled with galium-68 ([^68^Ga]Ga). The peptide AE105, consisting of nine amino acids, exhibits a strong binding affinity to the human uPAR protein. It forms a stable complex in a 1:1 stoichiometry, with a dissociation constant (KD) of 0.4 nM. AE105 has been found to be a highly effective competitive inhibitor of the uPA-uPAR interaction, with an inhibitory concentration (IC50) of 11 nM [22]. [^68^Ga]Ga has a known limitation in spatial resolution compared to other isotopes like copper-64 ([^64^Cu]Cu) [23]. [^64^Cu]Cu-uPAR-PET with [^64^Cu]Cu-DOTA-AE105 has only been evaluated in preclinical models in different cancers and in one phase I clinical trial in patients with breast, prostate, and lung cancer [24, 25] but never in head and neck cancer patients nor preclinical models that closely resembles OSCC tissue characteristics.

Most preclinical studies investigating imaging targets like uPAR have been performed in cell line-derived xenograft models. These models only partially mimic human malignancies and lack tumor heterogeneity and the cellular stromal tumor micro-environment-like cancer-associated fibroblasts and tumor-associated macrophages [26]. Patient-derived xenograft (PDX) models are models created by implantation of small and minimally processed patient-derived tumor pieces into immunodeficient mice. It has been demonstrated that these models preserve the tumor micro-environment, the heterogeneity, and mutations and have a high predictive value regarding patients [27, 28]. PDX models are thus expected to be a better and more realistic platform for developing new imaging modalities and therapy, especially for a target like uPAR, which is also expressed by tumor-infiltrating macrophages and fibroblasts in the tumor stromal compartment [29, 30]. PDX models of head and neck cancer have previously been studied and shown to be able to replicate human disease in terms of both histopathological and molecular characteristics [31–33]. In addition, PDX models of head and neck cancer have been demonstrated to mimic therapeutic response and have been proposed as a paraclinical model for investigating personalized therapy [34, 35]. To our knowledge, the expression of uPAR in PDX models has not previously been studied.

The primary aim of this study was to establish new OSCC PDX models, and the secondary goal was to investigate the use of [^64^Cu]Cu-uPAR-PET/CT in PDX models and evaluate the distribution of the tracer ([^64^Cu]Cu-DOTA-AE105) in tumor tissue.

## Materials and Methods

### Patient Selection and Study Design

**Fig. 1:**
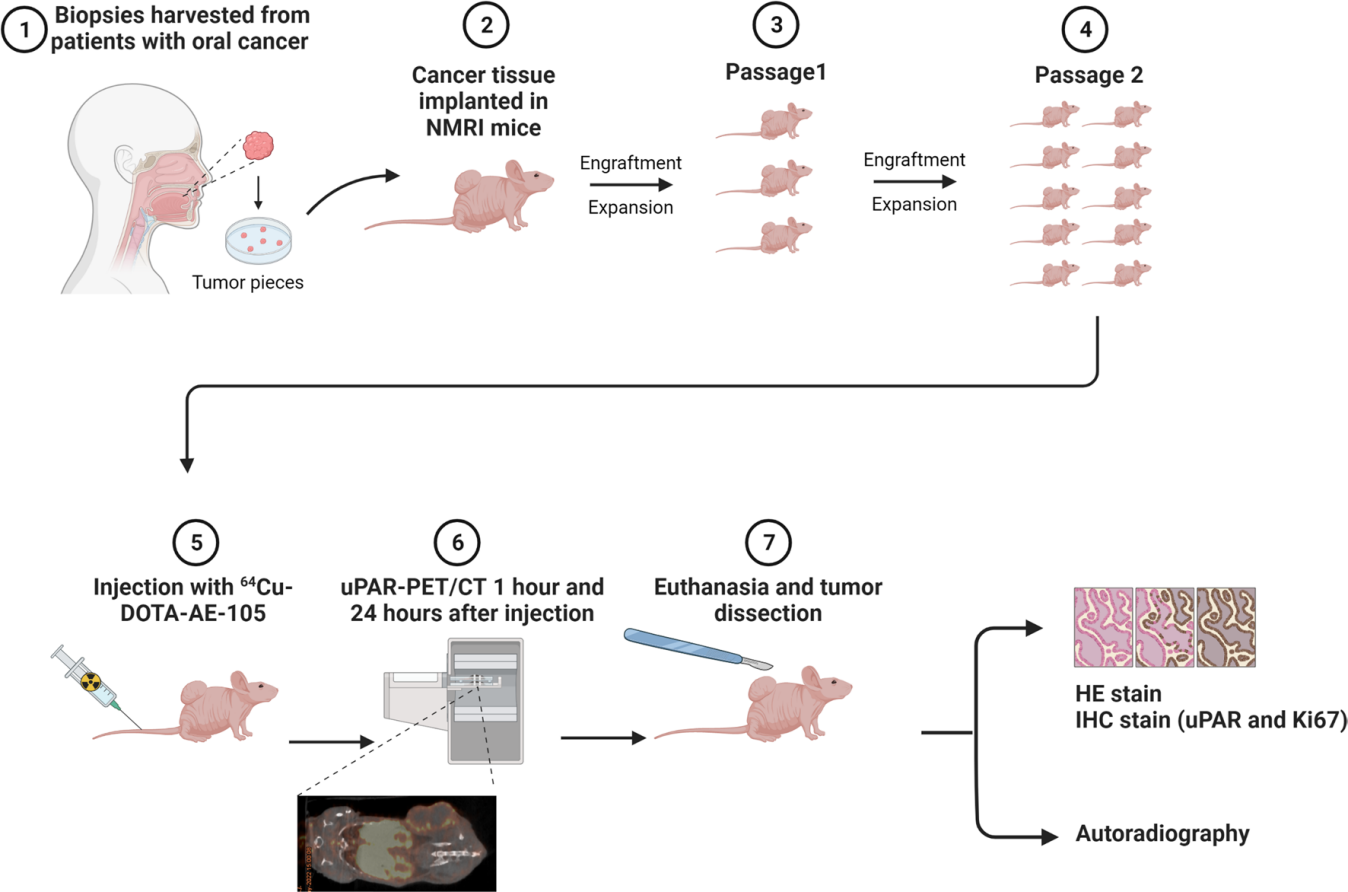
An overview of the study design showing the establishment of patient-derived xenograft models from three primary OSCC tumors, uPAR-PET/CT of 9–10 mice (passage 2) per tumor model followed by *ex vivo* analysis. (Created with BioRender.com)

Patients diagnosed with OSCC referred for primary surgery at the Department of Otolaryngology, Head and Neck Surgery and Audiology, Rigshospitalet between 2020 and 2021, were included and contacted regarding the donation of tumor tissue. Signed informed consent was obtained for all included patients. Clinicopathological data was collected from pathology reports. A biopsy was harvested from the resected primary tumor by a specialized head and neck pathologist (GL) without compromising tumor margin analysis. The primary tumor was divided into three pieces, one for formalin fixation, one for RNA analysis, and one for implantation in mice. The following implantation process and *ex vivo* analyses are shown in Fig. 1 and explained in detail below.

The study was approved by the Danish National Committee on Health and Research Ethics (H-17025452) and conducted in accordance with the Declaration of Helsinki (2002). Data were handled in accordance with the guidelines set by the Danish Data Protection Agency (No. 2012-58-0004)

### Animals

All animal experiments were performed in accordance with Danish laws under the license no. 2021-15-0201-01041. Female NMRI nude mice were used for the study (*n*=53 in total for establishment, expansion, and imaging study), Janvier Labs (Le Genest-Saint-Isle, France).

### Establishment of PDX Models

Within 1 h after the operation, tissue was implanted in mice. The pathologist localized viable tumor tissue in the resected specimen from primary tumor and placed the tissue in cold Gibco RPMI 1640 Media mixed with 10% Fetal Bovine Serum (Thermofisher Scientific, DK). Under aseptic conditions, tissue was chopped into a mesh, mixed 1:1 with Corning™ Matrigel™ (Thermofisher Scientific, DK) and divided in 5×5 mm samples for implantation in the flank of the mice (iteration 1, passage 0 (P0)). When tumor reached exponential growth, it was further passaged to 5 mice (P1) for expansion. Once tumors in P1 reached a volume > 1000 mm^3^, it was further passaged to 10–16 mice (P2) for use in the PET/CT study. The three models utilized were those that exhibited the highest degree of similarity in terms of growth and were available at the same time. The uPAR expression in these models was unknown at the time of inclusion. At date of PET scans, mice with tumors larger than 300 mm^3^ were included in the imaging study: model 1 (*n*=9), model 2 (*n*=10), and model 3 (*n*=10).

### Radiochemistry

The production of [^64^Cu]Cu-DOTA-AE105 is described in detail in supplementary material.

### Imaging Protocol and Imaging Analysis

Mice were anesthetized with 1.5% sevoflurane (Baxter Healthcare Ltd, UK) mixed with 35% O2 in ambient air through a nose cone, and 2 mice were scanned simultaneously. A dedicated small-animal PET/CT scanner (Inveon®, Siemens Medical Systems, PA, USA) were used. [^64^Cu]Cu -DOTA-AE105 was injected in a lateral tail vein and allowed to circulate in awake mice for 60 min before image acquisition. Images were analyzed as fused PET/CT images were circular regions of interest (ROIs) were drawn on CT images and superimposed on the fused PET image. ROIs were placed on every 4 slides in the axial plane on tumors and volumes calculated based on all ROIs in the tumor. Standardized uptake values (SUV), mean (SUV_mean_), and max (SUV_max_) were calculated for each tumor (more details in supplementary material).

### Autoradiography

Cryosections of 30 μm were cut on a Cryostat CM1860 (Leica Biosystems) with corresponding muscle and tumor samples placed on the same glass slide. Glass slides were covered with plastic foil and exposed for 1 h against a phosphor imaging plate (BAS-IP MS 2040E, GE Healthcare, MA, USA) in a light-shed cassette. Following exposure, phosphor imaging plates were analyzed using the Amersham Biomolecular Imager system (GE Healthcare, MA, USA) at a resolution of 10 μm.

### Immunohistochemistry

Formalin-fixated paraffin-embedded tumor samples from patients and the following PDX passages (0–2) were collected. Tumor samples from the P2 mice were gathered after the PET scan by sacrificing the animals. Three cross-sectional samples of the tumor were obtained and underwent hematoxylin and eosin (H&E) staining, Ki67 staining, and uPAR staining, respectively. All analysis was performed on 4 μm slides (see supplementary material for further details).

### Histology and Immunohistochemistry Evaluation

The biological stability of the PDX tumors was evaluated by a specialized head and neck pathologist by comparing the histological characteristics of the original patient tumor to matched tumor tissue from P0, P1, and P2. The following characteristics were evaluated: nuclear pleomorphism, stromal proportion, inflammatory cell infiltration, degenerative changes, the invasive front, proliferation ratio by Ki67 expression, and uPAR expression. All IHC-stained tumor samples were digitally scored using the open-source software Qupath [36]. For each sample, the tumor compartment, excluding necrosis and cystic regions, was digitally annotated. Positive and negative cells were digitally identified within the tumor compartment based on the mean DAB signal in the cell cytoplasm. Cell expansion was set to 5 μm, and intensity threshold was for uPAR and Ki67 set to 0.12 and 0.20 for weak intensity (+1), 0.25 and 0.40 for moderate intensity (+2), and 0.50 and 0.6 for strong intensity (+3), respectively. The H-score was digitally calculated for the annotated tumor compartments by adding 3 × percentage of strongly stained cells, 2 × percentage of moderately stained cells, and 1 × percentage of weakly stained cells, resulting in a score ranging from 0 to 300 [37].

### Statistical Analysis

Statistical analysis and bar charts were performed using GraphPad Prism version 9.3 for PC, GraphPad Software, La Jolla, CA, USA. To evaluate correlation between continuous variables, Pearson’s *R* squared test was applied. The unpaired *t*-test was used to determine the differences between two groups containing continuous variables. To evaluate tumor growth, we measured the number of days from implantation to tumor volume of 400 mm3. The tumor growth for the different PDX models was then visualized using Kaplan–Meier analysis. To determine the relationship between biomarker expression and tumor progression, the number of days from implantation to tumor volume of 400mm^3^ was correlated to H-scores of uPAR and Ki67. Continuous variables were reported as mean ± standard deviation (SD) or median with range. A *p*-value of less than 0.05 was considered statistically significant.

## Results

### PDX Models

PDX models were established from primary tumors of three patients with OSCC. The clinicopathological characteristics for alle donor patients are shown in Table 1. All three models were derived from aggressive tumors, which is reflected in their tumor characteristics, prognosis, and postoperative treatment. All three patients had stage III or stage IV disease with moderate to poor differentiation, non-cohesive invasion pattern, and perineural invasion, and two patients had vascular invasion. The patients were surgical treated with excision of primary tumor and neck dissection, followed by postoperative radiotherapy. Two patients experienced local recurrence within 1 year of the primary operation.

**Table 1:** Clinicopathological characteristics of the included OSCC patients

	Donor patients for PDX models		
Characteristics	Patient 1 (model 1)	Patient 2 (model 2)	Patient 3 (model 3)
Gender	Male	Male	Female
Age (years)	71	57	66
Tumor localization	Tongue	Floor of mouth	Floor of mouth
TNM stage (UICC 8)	T3N2bM0	T3N0M0	T2N2bM0
Depth of Invasion	8 mm	13 mm	10 mm
Perineural invasion	Yes	Yes	Yes
Vascular invasion	No	Yes	Yes
Pattern of invasion	Non-cohesive	Non-cohesive	Non-cohesive
HPV status	Negative	Negative	Negative
Histological grade of differentiation	Moderate	Poor	Moderate

For all models, we experienced an increasing tumor growth rate from initial implantation (P0) to the next passages. For the P2 models, the mean time from implantation to PET/CT was 67 days (range: 29–106 days). The median tumor size for P2 tumors at the time of PET/CT was 526 mm^3^ (range 301–1371 mm^3^).

### Histological Stability of PDX Model Tumors Compared to Patient Tumor

**Fig. 2:**
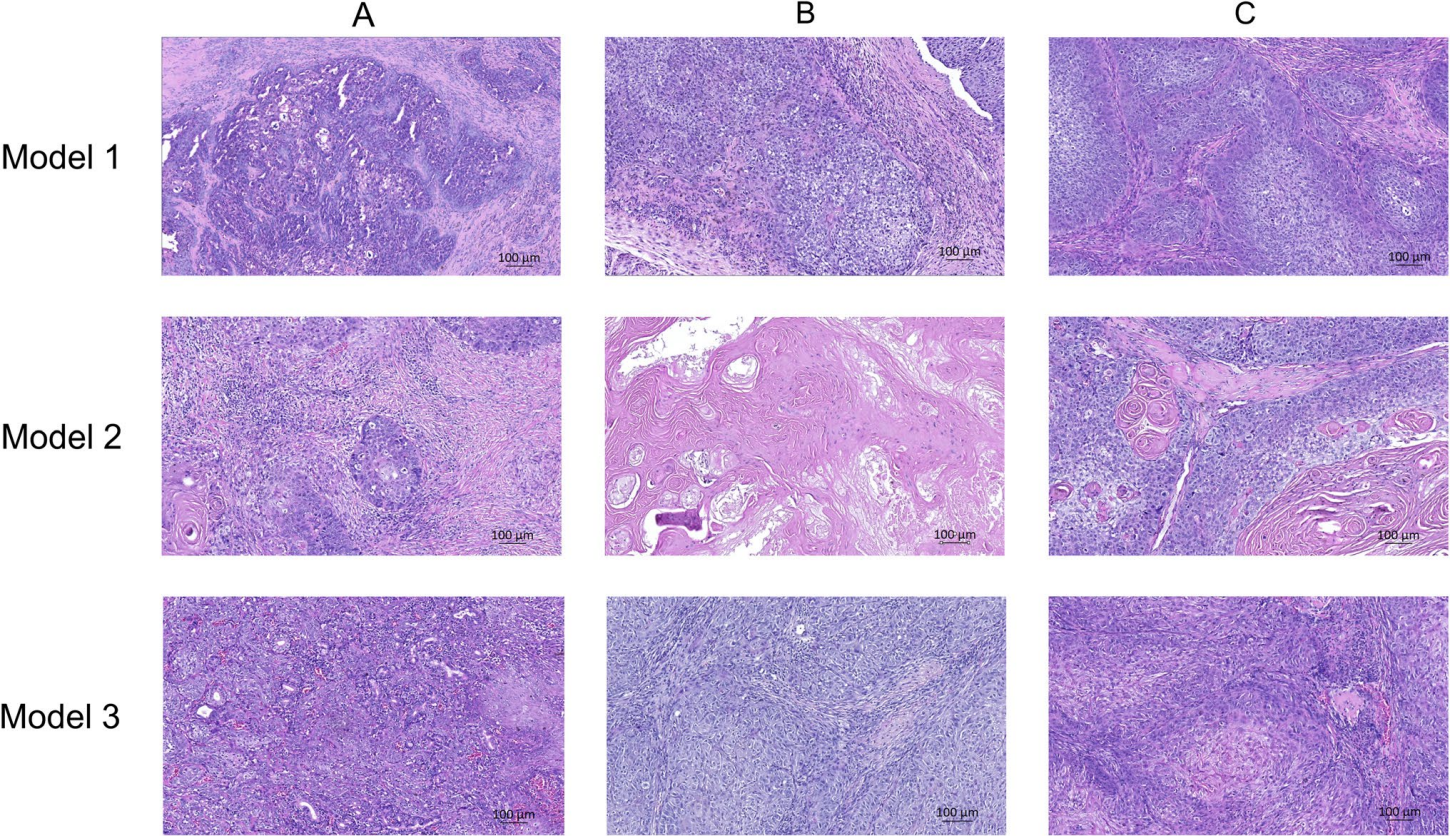
Representative samples of HE-stained tumor tissue from all three models at various passages demonstrating the histological stability in tumor tissue from the patient tumor to passage 2 PDX tumors. **A** Patient tumors. **B** Tumor from passage 1 PDX models. **C** Tumor from passage 2 PDX models

To ensure that tumors from PDX models resembled the primary tumor from patients, histopathological characteristics of donor tumors and subsequent passages were examined (Fig. 2). We found unchanged pleomorphism, grade of degenerative changes (cystic formation, focal necrosis, and keratinization), and pattern of the invasive front through the passages. Similarly, the expression of uPAR in the cytoplasm, membrane, and surrounding stroma of tumor cells remained unchanged from patient tumor to the different PDX models (P0–P2). In all models, uPAR was expressed more strongly in the invasive front and around necrosis/cysts. The extent of necrosis/cysts observed was limited in size and confined to a minority of tumors. In later passages, the tumor compartment contained a slightly reduced amount of stroma, a greater density of tumor cells, and less inflammatory cell infiltration. The tumors effectively capture the heterogeneity of primary tumors in terms of uPAR expression, as seen by the variable levels of uPAR expression observed in tumor tissue among mice within each model and across different PDX models (Table 2).

**Table 2:** Digital quantification of uPAR expression in IHC-stained tumor tissue from all the PET/CT-scanned mice. The varying staining intensity of tumor cells displays the heterogenous uPAR expression in tumor tissue across the different models. The H-score was digitally calculated by adding 1 × percentage of weakly stained cells, 2 × percentage of moderately stained cells, and 3 × percentage of strongly stained cells, yielding a score between 0 and 300

	PDX models		
uPAR expression	Model 1	Model 2	Model 3
Median H-score (range)	28.5 (11.5–96.5)	76.2 (58.8–98.2)	97.6 (70.7–127.7)
Median percentage of weakly stained tumor cells (range)	23.2 (10.1–65.9)	38.6 (20.8–44.1)	40.9 (39.2–50.9)
Median percentage of moderately stained tumor cells (range)	2.3 (0.6–13.7)	12.3 (8.5–18.7)	23.4 (11.9–36.2)
Median percentage of strongly stained tumor cells (range)	0.2 (0.1–1.1)	4.0 (1.9–9.4)	3.5 (0.7–8.5)

### Small-Animal PET/CT with [^64^Cu]Cu-DOTA-AE105

**Fig. 3:**
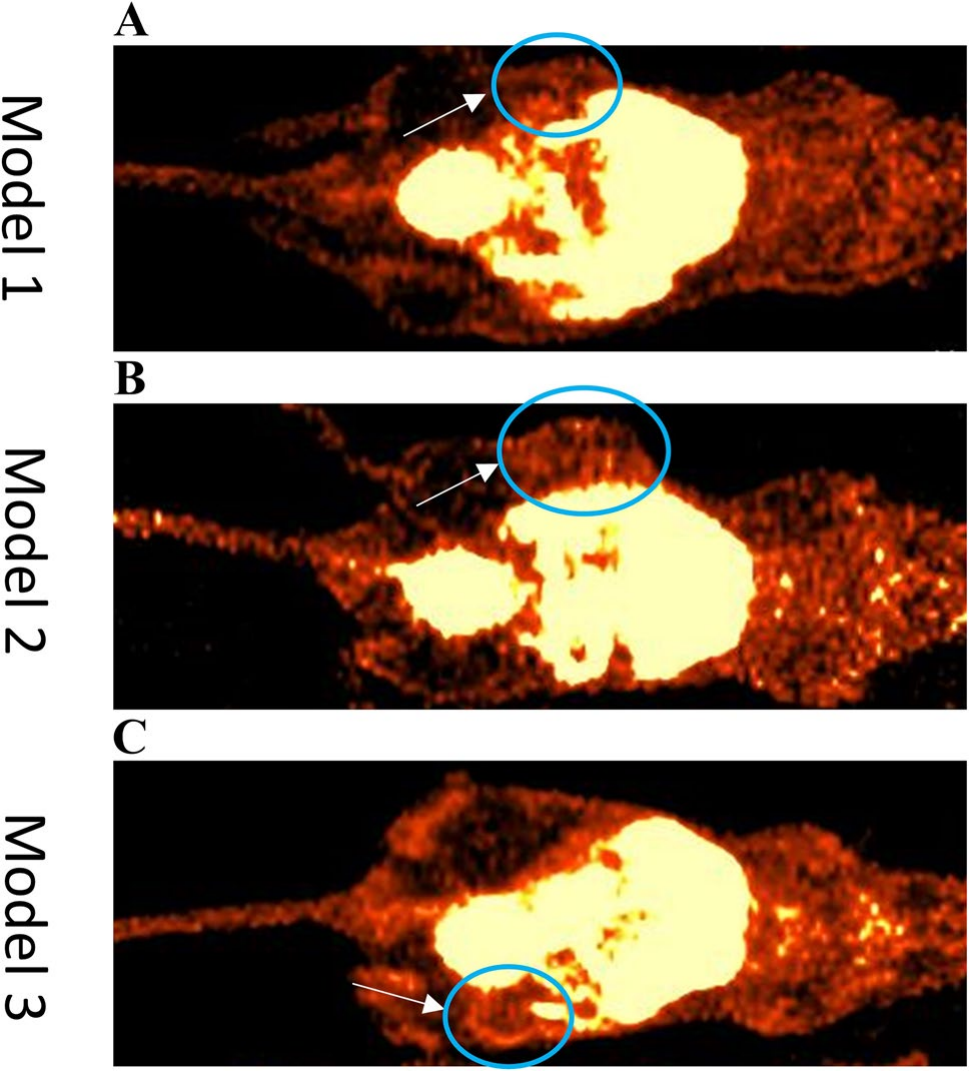
^64^Cu-uPAR-PET/CT 1 h after tracer injection in three OSCC PDX models. White arrows indicate tumor lesions, all of them located in the flank. **A** Model 1 with low diffuse uptake. **B** Model 2 with high diffuse uptake. **C** Model 3 showing high uptake with hot spots and rim enhancement around cystic tumor lesion

All models (29 mice) showed uptake of [^64^Cu]Cu-DOTA-AE105 in tumor compartment after both 1 h and 24 h. A heterogenic uptake pattern was seen in most tumors. A diffuse and lower uptake was seen in model 1 compared to the other models. Model 3 showed a generally higher uptake with hot spots in tumor tissue. Several tumors showed rim enhancement. Representative PET/CT images are shown in Fig. 3.

### Ex Vivo Validation of [^64^Cu]Cu-DOTA-AE105 Distribution Within Tumors

**Fig. 4:**
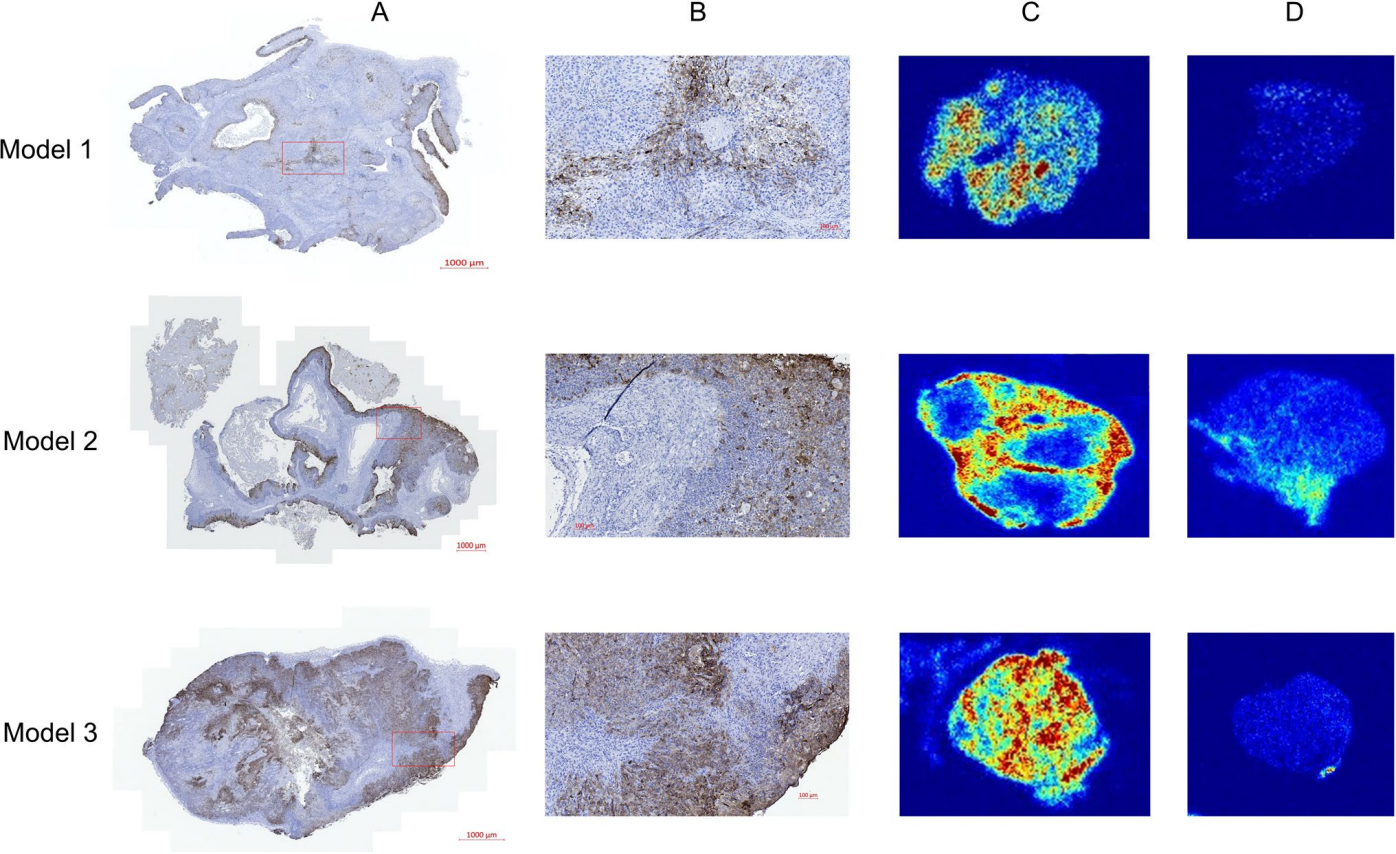
uPAR expression in tumor tissue by immunohistochemistry in each of the three PET/CT-scanned OSCC PDX models and autoradiography of the same tumors after tracer injection. This figure depicts the positive correlation between tumor regions exhibiting high tracer uptake and tumor regions with elevated levels of uPAR expression. **A** Microscopic image with low magnification of uPAR expression in tumor. **B** Microscopic section of uPAR expression showing both positive and negative cells. **C** Autoradiography from primary tumor. **D** Autoradiography from normal quadriceps muscle from the same mice

In Fig. 4, representative samples from each of the three PDX models show the uPAR expression determined by IHC staining in comparison to autoradiography of the same tumor. Topographically, the distribution of [^64^Cu]Cu-DOTA-AE105 in tumor tissue corresponds to the uPAR expression pattern revealed by IHC staining. In model 1 and model 3, a low and high expression was seen, respectively. In model 2, the IHC-stained cystic degeneration in the tumor compartment was also visible on the autoradiography sample. The muscle samples showed minimal [^64^Cu]Cu-DOTA-AE105 uptake on autoradiography for all PDX models confirming the low background uptake of the tracer and favorable tumor-to-muscle ratios.

**Fig. 5:**
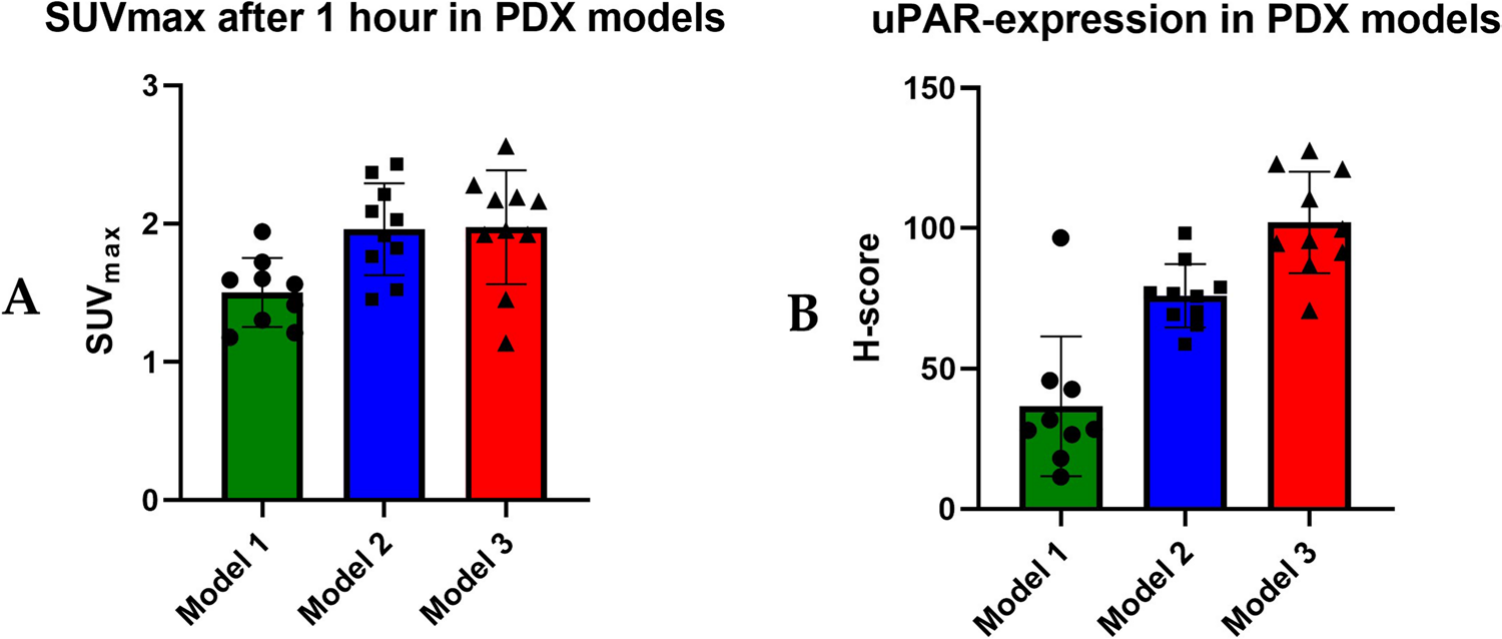
**A** Average SUV_max_ in tumor compartment for ^64^Cu-DOTA-AE105 PET/CT for three different PDX models (model 1 (*n*=9), model 2 (*n*=10), model 3 (*n*=10)). **B** Mean uPAR expression in tumor tissue quantified with H-score for the same three PDX models

All tumors expressed uPAR, but the H-score varied between the different models (Figs. 4 and 5A). The mean ± SD for SUV_max_ after 1 h for model 1, model 2, and model 3 was 1.50±0.24, 1.96±0.33, and 1.97±0.41, respectively. Significant difference in SUV_max_ values after 1 h was seen between model 1 and the two other models (*p*=0.0018). The same pattern was seen for uPAR expression (H-score) between the same models (*p*<0.0001) (Fig. 5B).

**Fig. 6:**
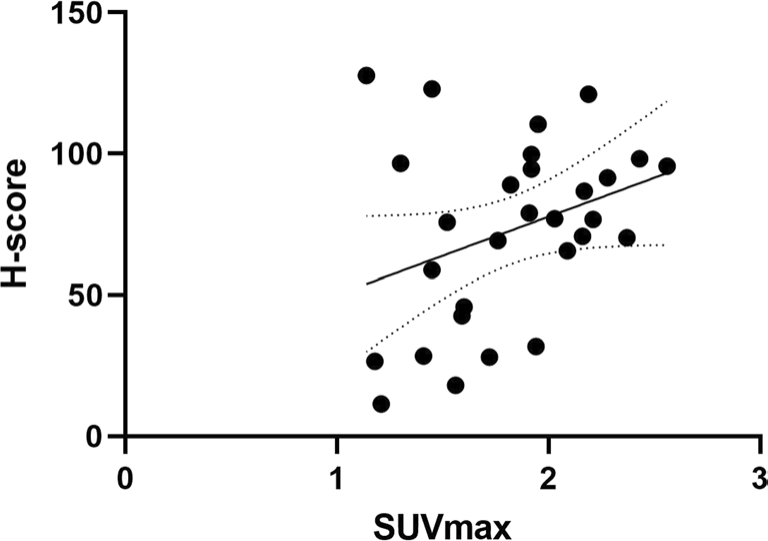
Correlation between ^64^Cu-uPAR-PET/CT SUV_max_ and uPAR expression, quantified with H-score, in tumor tissue from three different PDX models (*n*=29). No significant positive correlation was seen between SUVmax and H-score (*r*=0.34; *p*=0.07)

There was no significant positive correlation (*r* = 0.34; *p* = 0.07) between uPAR expression (H-score) and [^64^Cu]Cu-DOTA-AE105 tumor uptake after 1 h (SUV_max_) for all included tumor models (Fig. 6).

### Correlation Between Tumor Growth and uPAR Expression

We found a significant correlation between uPAR expression (H-score) in the tumor compartment and the number of days from implantation to tumor volume of 400 mm^3^ (*r*=−0.40, *p*=0.03), indicating that greater uPAR expression is associated with increased tumor growth rate (supplementary figure 1). No significant correlation was found between Ki67 expression and number of days from implantation to tumor volume of 400 mm^3^.

## Discussion

We successfully established three novel PDX models of OSCC by engrafting patient-derived tumor tissue from locally advanced OSCC into immunodeficient mice. Importantly, the models maintained histological stability, heterogeneity, uPAR expression, and Ki67 expression through passages. In these three OSCC PDX models, we studied the use of [^64^Cu]Cu-uPAR-PET/CT for imaging in a total of 29 tumors, a modality that has not previously been evaluated in head and neck cancer patients nor in heterogeneous preclinical PDX tumor models. We found a heterogenous tracer uptake in tumor tissue in all models. The tracer uptake and distribution in tumor were validated visually with autoradiography in comparison to IHC staining; however, quantitatively, there was no significant positive correlation between SUV_max_ and H-score. The lack of significant correlation may be due to the inherent limitations with IHC staining of tumor tissue which only samples a small section of the tumor, in contrast to PET imaging which captures the entire tumor. The tumor specificity of the tracer was demonstrated by the high autoradiography signal in tumor tissue compared to a minimal signal in samples from normal muscle. In addition, we observed a significant correlation between tumor growth and uPAR expression in the tumor compartment, demonstrating the prognostic potential of uPAR-targeted imaging in head and neck cancer.

These results indicate that OSCC PDX models can be used for investigating new molecular imaging modalities, such as [^64^Cu]Cu-uPAR-PET/CT, and might resemble human tumor tissue better than the more homogenous cell line xenograft models. Especially, when exploring targets like uPAR, which is expressed on tumor-associated activated stromal cells. Our findings regarding the histological stability of PDX tumor tissue through passages are consistent with previous studies, in which it has been demonstrated that histological properties, biomarker expression, and mutational profile are stable through passages [35, 38–40]. Therapeutic response to anti-cancer therapy in PDX models has also been shown to resemble the clinical response in matched patients [35, 40]. However, it has also been demonstrated that the human stromal composition is only maintained for early passages, after which the murine stroma dominates, suggesting that early passages may be better at resembling the donor tumor [41].

So far, only a few studies have investigated the use of uPAR-targeted PET imaging in head and neck cancer, but several studies have examined this imaging modality in other cancer types. In preclinical studies, [^64^Cu]Cu-labeled uPAR-targeting radioligands have been explored in different cancer cell line xenograft models [42, 43], and the correlation between [^64^Cu]Cu-DOTA-AE105 uptake in tumor and the uPAR expression was established by uPAR ELISA [43]. Other studies have investigated alternative chelators to DOTA [44] and established the dosimetry of [^64^Cu]Cu-DOTA-AE105 for planning clinical trials [45]. In humans, [^64^Cu]Cu-labeled PET imaging using [^64^Cu]Cu-DOTA-AE105 has so far only been investigated in a phase I clinical trial in 10 patients with breast, prostate, and bladder cancer demonstrating tumor uptake and providing evidence for safe use [24]. In OSCC, [^64^Cu]Cu-DOTA-AE105 has in a single preclinical cell line xenograft study demonstrated tracer-specific uptake in small orthotopic primary tongue tumors [25]. This current study is the first to study the use of [^64^Cu]Cu-DOTA-AE105 PET/CT in heterogenous, i.e., PDX, head and neck cancers tissue. The target specificity of this tracer has not previously been shown in PDX models with autoradiography nor with comparison between uPAR expression (quantified as H-score) and SUV_max_ value. The pronounced association between uPAR expression and [^64^Cu]Cu-DOTA-AE105 uptake found in this study has to our knowledge not previously been showed in cancer. These results emphasize the specificity of [^64^Cu]Cu-DOTA-AE105 PET/CT for imaging uPAR positive cancer tissue.

Another uPAR-PET tracer labeled with [^68^Ga]Ga ([^68^Ga]Ga-NOTA-AE105) has previously been investigated in head and neck cancer patients [21]. As the positron range for [^64^Cu]Cu (1mm) is shorter than for [^68^Ga]Ga (4mm), a [^64^Cu]Cu-labeled uPAR-PET tracer, like [^64^Cu]Cu-DOTA-AE105, may enhance detection of smaller tumor volumes as previously demonstrated by us in a head to head comparison of [^68^Ga]Ga- and [^64^Cu]Cu-labeled radiotracers targeting the somatostatin receptors in neuroendocrine tumors [23]. A tumor-specific imaging modality with high spatial resolution could have a significant impact on staging and treatment planning of patients with OSCC, particularly for those with early-stage disease with a high frequency of occult lymph node metastases. In addition, for studying tumor heterogeneity in a PDX model, the spatial resolution likewise is important.

We recently investigated the prognostic value of [^68^Ga]Ga-uPAR-PET/CT in 54 patients with head and neck cancer and found that high SUV_max_ values in primary tumor was significantly associated with poor survival and proposed this modality as a future tool for selecting patients to uPAR-targeted radionuclide therapy [21]. This theranostic concept has previously been demonstrated in both colorectal and prostate cancer cell line models using DOTA-AE105 radiolabeled with ^177^Lu for uPAR-targeted radionuclide therapy [9, 46]. Our OSCC PDX models, with a well characterized uPAR expression, could serve as a suitable translational platform for development of uPAR-targeted radionuclide therapy or other uPAR-targeted antitumor treatment strategies [47] for OSCC.

## Conclusions

We successfully established OSCC PDX models and demonstrated that their histological characteristics and uPAR expression closely resemble those of human tumors. [^64^Cu]Cu-uPAR-PET/CT showed target- and tumor-specific uptake in OSCC PDX models demonstrating the diagnostic potential of this modality for OSCC patients. In addition, we found that uPAR expression in OSCC PDX tumors was correlated with tumor growth rate emphasizing the prognostic potential of this biomarker. These findings suggest that OSCC PDX models could serve as a valuable preclinical platform for evaluating uPAR-targeted molecular imaging and therapy modalities.

### Supplementary Information

ESM 1 (DOCX 77 kb)
