# Supplementary material for: Urokinase-Type Plasminogen Activator Receptor (uPAR) Expression and [64Cu]Cu-DOTA-AE105 uPAR-PET/CT in Patient-Derived Xenograft Models of Oral Squamous Cell Carcinoma
Source: Mol Imaging Biol. 2023 Sep 25;25(6):1034–44. doi: 10.1007/s11307-023-01858-x (PMC10728257; doi:10.1007/s11307-023-01858-x)
Supplement: Supplementary file 1 — (DOCX 77 kb) [file 11307_2023_1858_MOESM1_ESM.docx]

**Electronic Supplementary Material**

**Title:**

Urokinase type plasminogen activator receptor (uPAR) expression and ^64^Cu-uPAR-PET/CT in patient derived xenograft models of oral squamous cell carcinoma

**Journal:** Molecular Imaging and Biology

**Authors:**

Mads Lawaetz^1,2^, Tina Binderup^2^, Anders Christensen^1^, Karina Juhl^2^, Giedrius Lelkaitis^3^, Eva Lykke^1^, Line Knudsen^2^, Christian von Buchwald^1^ and Andreas Kjaer^2^*****

**Affilitation:**

1. Department of Otolaryngology, Head and Neck Surgery and Audiology, Rigshospitalet, Copenhagen University Hospital, Copenhagen, Denmark
2. Department of Clinical Physiology, Nuclear Medicine and PET and Cluster for Molecular Imaging, Copenhagen University Hospital - Rigshospitalet & Department of Biomedical Sciences, University of Copenhagen, Copenhagen, Denmark
3. Department of Pathology, Rigshospitalet, Copenhagen University Hospital, Copenhagen, Denmark

**Corresponding author**: Mads Lawaetz, MD, E-mail: madslawaetz@gmail.com

**Radiochemistry:**

All commercially available chemicals and reagents were used without further purification. [^64^Cu]Cu was obtained as dry [^64^Cu]CuCl_2_ (~1 GBq) from Hevesy Laboratory, DTU Nutech Risø. Analytical radio high-performance liquid chromatography (HPLC) was performed on a Dionex Ultimate 3000, using a Phenomenex Luna 5 µm C18(2) 100 Å 150 x 4.6 mm column, a flow rate of 1.5 mL/minutes, a gradient going from 0% to 100% B in A (H_2_O/MeCN/TFA, A 100/0/0.1, B 0/100/0.1) in 10 minutes, and an injection volume of 20 µL.

DOTA-AE105 was radiolabeled with [^64^Cu]Cu to obtain [^64^Cu]Cu -DOTA-AE105. First, dry [^64^Cu]CuCl_2_ was dissolved in 70 µL TraceSelect water and left for 15 minutes with periodic gentle shaking. Ammonium acetate buffer with gentisic acid was prepared by dissolving 77 mg ammonium acetate in TraceSelect water (~9 mL), adjusting the pH to 8.4 ± 0.1 with 2 N NaOH, adding 50 gentisic acid and adjusting the final volume to 10 mL with TraceSelect water, the final pH should be 5.2 ± 0.1 and the solution should be clear. Labeling was performed by adding 50 µL of [^64^Cu]CuCl2 (~360 MBq) to a vial containing 3 µL DOTA-AE105 (2 mg/mL) and 450 µL of the prepared ammonium acetate buffer with gentisic acid. The reaction mixture was incubated for 5 minutes at 80°C. After the reaction a small sample was retrieved and diluted for radio-HPLC analysis. Purification was performed by diluting the reaction mixture in 10 mL MilliQ water and passing the solution through a Sep-Pak C18 Plus Light cartridge (Waters) preconditioned with 5 mL EtOH and 5 mL MilliQ water. The cartridge was eluted with 0.5 mL EtOH into a vial containing 9.5 mL PBS yielding the final formulated tracer. An undiluted sample was retrieved for radio-HPLC analysis.

The labeling of DOTA-AE105 with [^64^Cu]Cu was achieved in 76%-78% decay corrected radiochemical yield with a radiochemical purity of >99%, as determined by radio-HPLC, in a total synthesis time of 45 minutes. The molar activity of the final PBS formulated tracer was 53-64 MBq/nmol with a radioactive concentration of 37-39 MBq/mL.

**Imaging protocol and imaging analysis:**

Mice were anesthetized with 1.5% Sevoflurane (Baxter Healthcare Ltd, UK) mixed with 35% O2 in ambient air through a nose cone and 2 mice were scanned simultaneously. A dedicated small-animal PET/CT scanner (Inveon®, Siemens Medical Systems, PA, USA) were used with the CT settings: Exposure time 400 msec, voltage 65 kVp and tube current 500 μA with 180 projections. CT images were reconstructed using an Feldkamp cone beam algorithm.

[^64^Cu]Cu -DOTA-AE105 was injected in a lateral tail vein and allowed to circulate in awake mice for 60 minutes before image acquisition. The dose was 5.31 ± 0.34 MBq (mean ± SD) and image acquisition time 10 minutes in a 1 bed position. Mice were scanned at 1 hour and 24 hours post injection.

Scatter and attenuation corrected was applied and PET images reconstructed using the OSEM3D/MAP algorithm.

Images were analyzed as fused PET/CT images using the Inveon Software (Siemens Medical Systems, PA, USA) were circular Regions of Interest (ROIs) were drawn on CT images and superimposed on the fused PET image. ROIs were placed on every 4 slides in the axial plane on tumors, and volumes calculated based on all ROIs in the tumor. Standardized Uptake values (SUV), mean (SUV_mean_) and max (SUV_max_) were calculated for each tumor: SUV = (Uptake in tumor volume)(decay corrected injected dose)/(weight of the mouse).

**Immunohistochemistry:**

The expression of Ki67 and uPAR was determined by immunohistochemistry on 4 μm slides. For both stains, slides were incubated at 60°C for 60 minutes, then deparaffinized with xylene and rehydrated using alcohols of decreasing concentration.

For uPAR, antigen retrieval was performed for 10 minutes at 95°C in CC1 antigen retrieval buffer (Ventana Medical Systems). For Ki67, antigen retrieval was performed for 15 minutes at 95 °C in pH 6 buffer. For both biomarkers, endogenous peroxidase was inhibited by peroxidase-blocking solution for 8 minutes (DAKO s2023). Two percent BSA was utilised to inhibit non-specific antibody binding.

For uPAR and Ki67, slides were incubated for one hour with uPAR-specific antibody (GeneTex product no. GTX100467, concentration 1:500) and Ki67 specific antibody (Abcam product no. Ab15580, concentration 1:500), respectively. For both biomarkers, the slides were subsequently incubated for 45 minutes with the secondary antibody (DAKO Anti-rabbit K4003). The staining was visualized with a DAB+ substrate chromogen system (DAKO K3468) and then hematoxylin was applied for 60 seconds.

**Supplementary figure 1:**


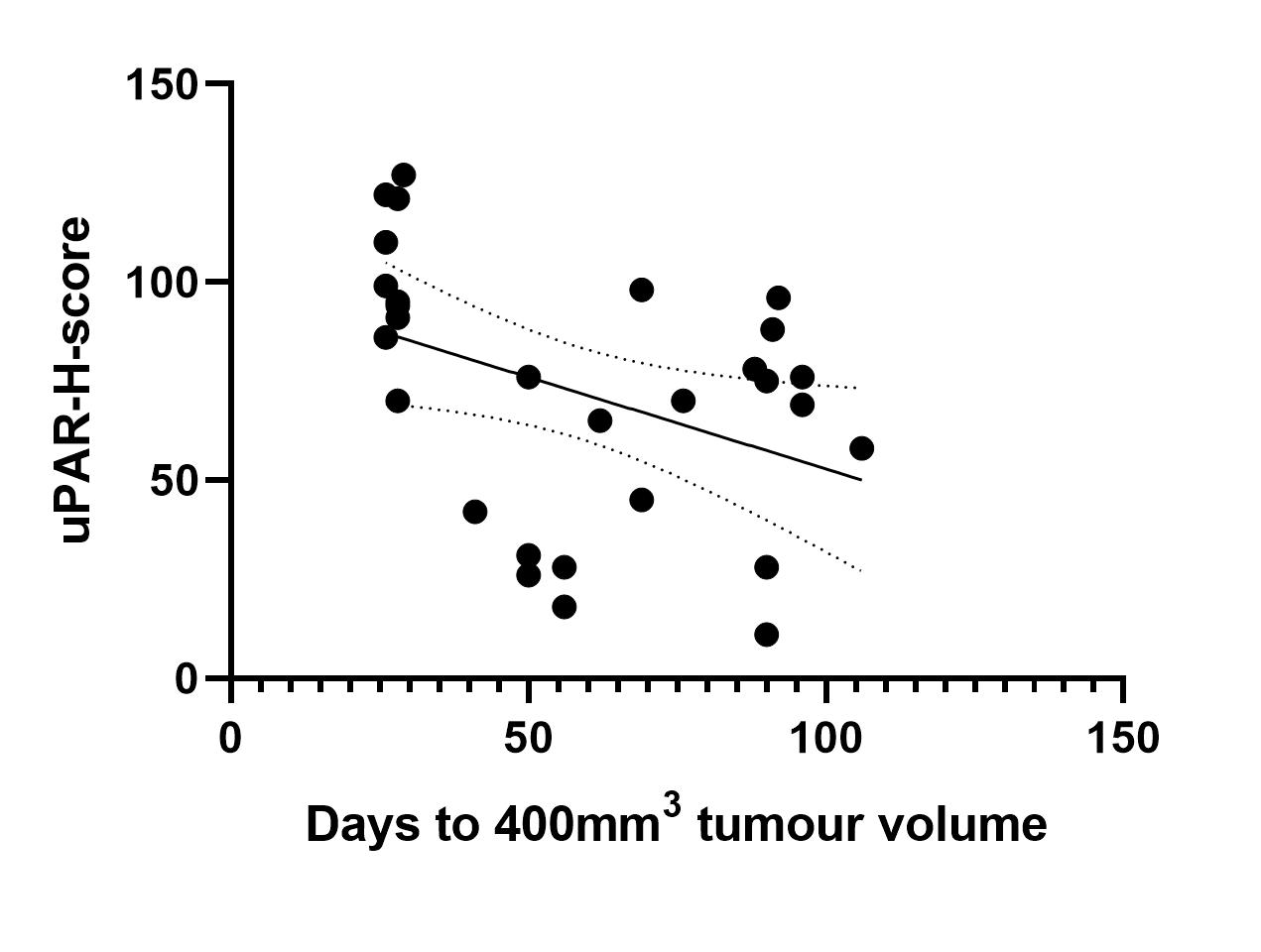


**Supplementary figure 1:** Correlation between uPAR expression quantified with H-score and tumor growth measured as number of days from implantation of tumor in 29 mice until the tumor reached 400 mm3 (r=-0.40; p=0.03).
